# Validation of six commercially available angiotensin II type 1 receptor antibodies: AT1R antibody validation

**DOI:** 10.3724/abbs.2024199

**Published:** 2025-01-07

**Authors:** Bingjie Li, Xi Zhang, Fei Sun, Xingzhong Zhang, Huirong Liu, Suli Zhang

**Affiliations:** 1 Department of Physiology & Pathophysiology School of Basic Medical Sciences Capital Medical University Beijing 100069 China; 2 Beijing Key Laboratory of Metabolic Disorder Related Cardiovascular Disease Capital Medical University Beijing 100069 China; 3 State Key Laboratory of Cardiovascular Diseases Fuwai Hospital Chinese Academy of Medical Sciences Beijing 102308 China; 4 Laboratory for Clinical Medicine Capital Medical University Beijing 100069 China

The renin-angiotensin system (RAS) is a crucial regulatory mechanism for cardiovascular function [1]. The angiotensin II (Ang II) type 1 receptor (AT1R) is the principal receptor responsible for mediating RAS function. AT1R belongs to the G protein-coupled receptor (GPCR) family and is present in multiple tissues, including vascular smooth muscle, endothelium, heart, brain, kidney, adrenal gland, and adipose tissue [2]. Physiologically, AT1R mediates second messenger signaling through classical G proteins. Ang II binding to AT1R predominantly activates Gq/11, leading to the activation of phospholipase C (PLC), which results in the production of inositol 1,4,5-trisphosphate (IP3) and diacylglycerol (DG). Then, increased Ca ^2+^ is released from the sarcoplasmic reticulum to mediate the processes of vasoconstriction, enhance cardiac contractility, regulate water‒salt balance, *etc*. [3]. Under pathological conditions, AT1R aberrantly activates G proteins, including mitogen-activated protein kinases (MAPKs: ERK1/2, JNK, and p38MAPK), receptor tyrosine kinases (PDGF, EGFR, and insulin receptor), non-receptor tyrosine kinases [Src, JAK/STAT, and focal adhesion kinase (FAK)], and NADPH oxidase, to influence downstream pathways. This activation exacerbates inflammatory responses, fibrosis, and pathological cardiovascular remodeling [4]. AT1Rs within the nervous system can also induce excessive activity in the sympathetic nervous system, which increases myocardial strain and facilitates the progression of heart failure [5].

Owing to the importance of AT1R in a variety of diseases, greater demands have been placed on the accuracy of AT1R detection. The structural complexity and low immunogenicity of GPCRs pose considerable challenges in the development of specific antibodies. Many commercially available antibodies for GPCRs, such as those against muscarinic and adrenergic receptors, lack specificity [6]. Current studies on AT1R often use these commercial antibodies, but many fail to demonstrate specificity when *AT1R*-knockdown or AT1R-overexpressing tissues and cells are tested [7].

This study aims to specifically validate six newly available commercial AT1R antibodies ( Supplementary Table S1). Using *AT1R* global knockout SD rats, cardiomyocyte conditional *AT1R* knockout C57BL/6N mice, AT1R-overexpressing CHO stable-transformed cell lines and AT1R-overexpressing HEK293 cells, we assessed AT1R expression and localization through receptor-ligand binding assays, RT-PCR, western blot analysis, and immunocytochemistry. Materials and methods are available in Supplementary Materials and Methods.

To verify the specificity of the antibody, we generated *AT1R*-global knockout SD rats ( *AT1R*-KO) using CRISPR-Cas9 technology. Agarose gel electrophoresis revealed bands at approximately 470 bp for *AT1R*-KO rats and 531 bp for wild-type (WT) rats, confirming successful *AT1R* knockout at the gene level ( Figure 1A). RT-PCR analysis of vascular tissue RNA revealed the absence of AT1R in *AT1R*-KO rats ( Figure 1B). Ligand-receptor binding assays revealed significantly less ^125^I-Ang II binding to vascular tissue proteins in *AT1R*-KO rats than in WT rats ( Figure 1C). Additionally, primary cardiomyocytes extracted from 0–3-day-old WT and *AT1R*-KO neonatal rats presented a significant increase in beating rate upon Ang II stimulation in WT rats, whereas no response was observed in *AT1R*-KO rats ( Figure 1D). These results confirmed successful *AT1R* global knockout in AT1R-KO rats.

*AT1R*-KO rats were thus utilized to verify the specificity of AT1R antibodies (A14201, 25343-1-AP, and 66415-1-Ig). Western blot analysis was conducted on protein extracts from the heart, vascular, liver, and kidney tissues of WT and *AT1R*-KO rats. Under room temperature denaturation conditions, the A14201 antibody detected AT1R bands at the expected molecular weight (42 kDa) in all tissues from WT rats, the 25343-1-AP antibody detected AT1R in heart and kidney tissues, and the 66415-1-Ig antibody detected AT1R only in the heart tissues. Compared with WT control rats, the A14201 antibody revealed a reduction in AT1R protein expression in AT1R-KO rats across all tissues, whereas 25343-1-AP and 66415-1-Ig did not significantly change AT1R protein expression in *AT1R*-KO rats ( Figure 1E). Under high-temperature denaturation conditions, A14201 detected AT1R in all tissues from WT rats, 25343-1-AP detected AT1R in the heart and liver tissues, and 66415-1-Ig failed to detect AT1R in any tissue. However, the differences in AT1R expression between WT and *AT1R*-KO rats were not significant under these conditions according to the use of any antibody ( Figure 1F). These findings suggested that the A14201 antibody has better specificity for detecting AT1R expression in various rat proteins under room temperature denaturation conditions in western blot analysis experiments.

We further validated available commercial AT1R antibodies in *AT1R*-conditional knockout ( *AT1R*-CKO) cardiomyocytes generated using Cre-loxP recombination technology. Agarose gel electrophoresis confirmed the presence of Flox bands at 335 bp and Cre bands at 300 bp in *AT1R*-CKO mice ( Figure 1G). RT-PCR analysis of cardiomyocyte RNA revealed the absence of AT1R in *AT1R*-CKO mice ( Figure 1H).

*AT1R*-CKO mouse cardiomyocytes and kidney tissue at the protein level were further utilized to detect antibody specificity (A14201, 25343-1-AP, 66415-1-Ig, PA5-18587, GTX89149, and ab124505). Western blot analysis of cardiomyocyte proteins under denaturation at room temperature revealed that A14201, 25343-1-AP, and ab124505 antibodies detected AT1R bands at 42 kDa in the WT mouse group, with reduced AT1R expression in the *AT1R*-CKO mouse group. However, there was no significant change in AT1R expression detected by the GTX89149 antibody compared with that in the control group. 66415-1-Ig and PA5-18587 antibodies failed to detect significant AT1R bands ( Figure 1I). Under high-temperature denaturation conditions, AT1R bands were detected for A14201, 25343-1-AP, 66415-1-Ig, PA5-18587, and ab124505, whereas GTX89149 presented no significant bands at 42 kDa. Reduced AT1R expression in *AT1R*-CKO cardiomyocytes was detected by A14201 and 25343-1-AP antibodies but not by 66415-1-Ig, PA5-18587, or ab124505 antibodies ( Figure 1J). For mouse kidney proteins, western blot analysis revealed that A14201 and PA5-18587 antibodies can detect AT1R bands under either room temperature or high-temperature denaturation conditions. No significant changes in AT1R expression were observed between WT and *AT1R*-CKO mouse kidney tissues after denaturation at room temperature. AT1R expression was inconsistent between the two groups under high-temperature denaturation. However, the 25343-1-AP, 66415-1-Ig, GTX89149 and ab124505 antibodies failed to detect significant AT1R bands ( Figure 1K,L). The data presented herein provide evidence that the A14201 antibody can specifically detect the expression of AT1R in mice, as determined by western blot analysis.

To evaluate the specificity of antibodies in detecting AT1R in cells, we used the human AT1R sequence to overexpress AT1R in CHO cells (AT1R-CHO) and AT1R in HEK293 cells (AT1R-HEK293). Western blot analysis of AT1R-CHO under room temperature denaturation conditions revealed that A14201 and 25343-1-AP antibodies detected AT1R bands at 42 kDa, whereas the 66415-1-Ig, PA5-18587 and GTX89149 antibodies were unable to detect any bands. The A14201 antibody increased AT1R protein expression in AT1R-CHO under room temperature denaturation conditions, whereas the 25343-1-AP antibody detection of AT1R expression was consistent between the two groups. Under high-temperature denaturation conditions, western blot analysis revealed that A14201 and PA5-18587 antibodies can detect AT1R bands at 42 kDa. However, the 25343-1-AP antibody detected AT1R bands at 42 kDa only in the AT1R-CHO group but not in the WT group. 66415-1-Ig and GTX89149 were unable to detect any bands. The A14201 antibody increased AT1R expression in AT1R-CHO cells under high-temperature denaturation conditions, whereas the GTX89149 antibody decreased AT1R expression in AT1R-CHO cells ( Figure 1M,N). For AT1R-HEK293 cells, the detection efficiency of the commercially available AT1R antibody was not the same as that of CHO cells. Under conditions of denaturation at room temperature and high temperature, western blot analysis revealed that A14201 and 25343-1-AP antibodies can detect AT1R bands in HEK293 cells. Interestingly, only the A14201 antibody increased AT1R expression in AT1R-HEK293 cells compared with that in HEK293 cells under room temperature denaturation conditions, but not under high-temperature denaturation conditions or with the 25343-1-AP antibody. Conversely, no bands were detected by the 66415-1-Ig, PA5-18587 or GTX89149 antibodies ( Figure 1O,P). These results showed that the A14201 antibody is specific for detecting AT1R expression in cells via a western blot analysis.

The specificity of commercially available AT1R antibodies was further assessed via immunocytochemistry assays on cardiomyocytes from 0–3-day-old WT and *AT1R*-KO SD neonatal rats, as well as normal CHO cells and AT1R-CHO cells. All the antibodies produced positive fluorescent signals, but there was no significant difference in fluorescence intensity between the WT and *AT1R*-KO rats ( Figure 2A) or between normal CHO and AT1R-CHO cells ( Figure 2B). These findings indicate that AT1R antibodies lack specificity in immunocytochemistry assays for detecting AT1R expression.

In this study, we demonstrated that commercially available AT1R antibodies exhibited variable immunoreactivity across different tissues and cells. We tested the effectiveness of six common AT1R antibodies, including five anti-human, three anti-rat, and six anti-mouse AT1R antibodies, under both room temperature and high-temperature denaturation conditions. The results indicated that in the western blot analysis, room temperature denaturation was more effective at detecting AT1R expression in the *AT1R*-knockdown and AT1R-overexpressing models. The A14201 antibody showed the highest specificity in distinguishing *AT1R* knockdown and overexpression across different species. However, in immunocytochemical experiments, the fluorescence intensity of the *AT1R* knockout or AT1R-overexpressing groups was unchanged compared with that of their respective controls; in other words, all the antibodies lacked specificity.

The renin-angiotensin system (RAS) is crucial for maintaining homeostasis and regulating blood pressure, renal function, sympathetic nervous system activity, and cardiovascular health. AT1R, a key receptor in the RAS, is implicated in various physiological and pathological responses. Acute AT1R activation initially helps maintain cardiovascular function under stress, but prolonged overstimulation exacerbates oxidative stress, cardiac remodeling, and dysfunction, leading to maladaptive hypertrophy and heart failure [8]. Therefore, accurate detection of AT1R expression and localization is imperative.

AT1Rs belong to the class of G protein-coupled receptors with seven transmembrane domains. It is expressed in cardiac myocytes and has potential actions such as inducing cellular hypertrophy and activating pathways associated with fibrosis and inflammation. Humans have one isoform of AT1R, whereas rodents have two (AT1aR and AT1bR) that share 94% identity [9].

AT1aR is the dominant type in mice, and studies have focused on its role. Existing studies using AT1aR-deficient mice suggested that blood pressure elevation and after-load play predominant roles in driving Ang II-dependent cardiovascular disease. A cardiomyocyte-specific *AT1aR* knockout mouse model was generated to study the role of cardiomyocyte AT1R in cardiovascular diseases, but the detection of AT1R presents difficulties due to the high amino acid similarity, posing a challenge for antibodies designed to detect endogenous AT1R proteins. However, many commercially available antibodies are used to study AT1R expression, localization, and regulation across various species, tissues, and cells. Our results showed that the A14201 antibody effectively detected changes in AT1R expression under room temperature denaturation but not under high-temperature conditions ( Figure 1E,F,I,L,M–P). Excessive protein structure disruption caused by high-temperature denaturation likely impairs the ability of antibodies to differentiate AT1Rs from similar proteins, underscoring the importance of maintaining the spatial structure of proteins for accurate antibody detection. However, immunocytochemical staining ( Figure 2A,B) showed no significant difference in fluorescence intensity between the knockout or overexpression groups and their controls, indicating a lack of specificity of the existing antibodies.

Antibody preparation methods include the use of polyclonal antibodies from conventional animal immunization, monoclonal antibodies from hybridoma technology, and genetically engineered antibodies. Monoclonal antibodies are produced by immunizing mice with antigens, fusing splenic B cells with tumor cells to create hybridomas, and then expanding and purifying the antibody-producing cells. Generally, the specificity of monoclonal antibodies is superior to that of polyclonal antibodies [10], which may also be one of the reasons why A14201 antibodies are most effective.

Despite advances such as determining the crystal structure of human AT1R via continuous femtosecond crystallography, the structural and amino acid similarities of AT1R isoforms introduce variability in antibody specificity. Selecting specific fragments is crucial for developing effective antibodies. We screened the immunogens for antibodies. The A14201 antibody was generated using the recombinant fusion protein of the AT1R amino acid sequence 265–359 (95 aa) as the immunogen. The 25343-1-AP and 66415-1-Ig antibodies were generated using the recombinant fusion protein of the AT1R amino acid sequence 290–359 (70 aa) as the immunogen. The PA5-18587 and GTX89149 antibodies were generated using the peptide of the AT1R amino acid sequence 227–240 (14 aa) as the immunogen, while the immunogen for the ab124505 antibody was not disclosed. On the basis of the above immunogens, we found that the A14201 antibody has the longest immunogen, from which we infer that antibodies produced from a longer amino acid sequence may exhibit greater specificity.

We used Clustal Omega software for sequence comparison and revealed that the homology of AT1R between mice and humans was 94.43%, whereas that between rats and humans was 94.71% ( Table 1). These findings indicated that human AT1R is highly homologous to AT1R in mice and rats. This finding further explains the specificity of the A14201 antibody in detecting AT1R expression in rats, mice and cells.

**Table:** **Table 1** Comparison of the full protein sequences of AT1R as well as the amino acid sequences from positions 265 to 359 of AT1R in humans, mice, and rats

AT1R sequence	Description	Score	Score	Score
AT1Rtotal	sp|P30556|AGTR1_HUMAN	100.00	94.43	94.71
sp|P29754|AGTRA_MOUSE	94.43	100.00	98.61
sp|P25095|AGTRA_RAT	94.71	98.61	100.00
AT1R265–359	sp|P30556|AGTR1_HUMAN	100.00	89.47	90.53
sp|P29754|AGTRA_MOUSE	89.47	100.00	96.84
sp|P25095|AGTRA_RAT	90.53	96.84	100.00

AT1Rtotal, total protein sequence of AT1R; AT1R265–359, the 265 to 359 amino acids of AT1R.

In conclusion, while the A14201 antibody is specific for AT1R detection in western blot analysis under room temperature denaturation conditions across various species and cell lines, all commercially available antibodies lack specificity in immunocytochemistry assays. Therefore, multiple methods are needed to ensure accurate results.

**Figure 1:**
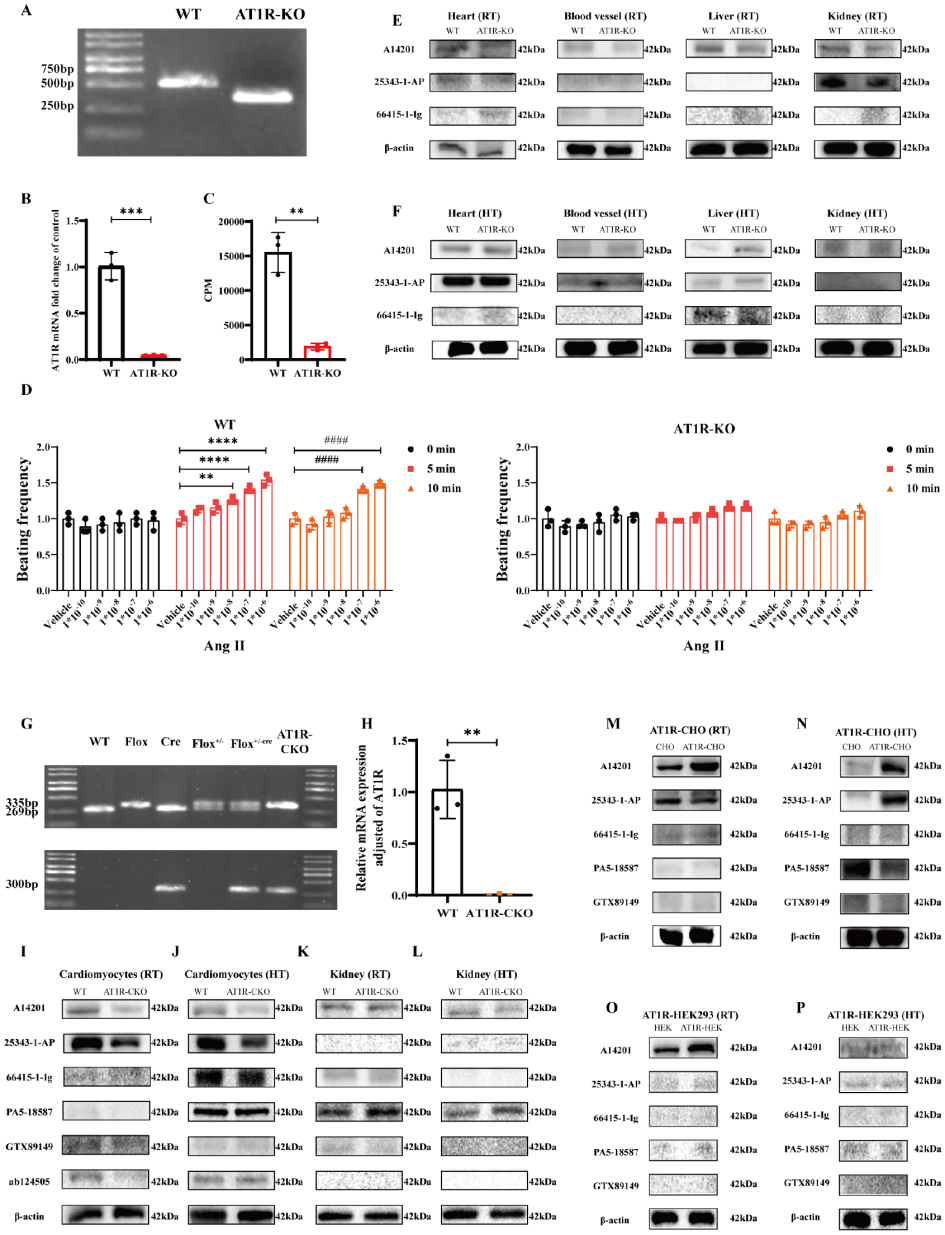
Figure 1 Evaluation of six commercially available angiotensin II type 1 receptor antibodies by western blot analysis (A) AT1R-global knockout SD rats were screened using agarose gel electrophoresis, which revealed a 470-bp AT1R-KO rat band and a 531-bp WT rat band. (B) RT-PCR analysis of AT1R mRNA expression in the blood vessels of wild-type and AT1R-KO rats, normalized to β-actin, indicated a significant difference ( P = 0.0004, WT vs AT1R-KO; n = 3). (C) Ligand receptor binding assay detecting 125I-Ang II binding also showed a significant difference (P = 0.0013, WT vs AT1R-KO; n = 3). (D) Cardiomyocyte extraction from wild-type and AT1R-KO neonatal rats, followed by stimulation with varying concentrations of Ang II to observe cardiomyocyte beating, revealed significant differences (P = 0.0048, vehicle vs 1×10–8 Ang II; **P < 0.01, vehicle vs 1 × 10–9/1 × 10–10 Ang II; #### P < 0.0001, vehicle vs 1 × 10–9/1 × 10–10 Ang II; n = 3). (E,F) Western blot analysis of AT1R expression in proteins extracted from heart, blood vessels, liver, and kidney tissues of WT and AT1R-KO rats under room temperature denaturation (E) or high temperature denaturation (F) conditions. (G) AT1R-CKO mice were screened via agarose gel electrophoresis, showing Flox bands at approximately 335 bp and cre bands at approximately 300 bp. (H) RT-PCR analysis of AT1R mRNA expression in cardiomyocytes of WT and AT1R-CKO mice, normalized to β-actin, showed a significant difference (P = 0.0034, WT vs AT1R-CKO; n = 3). (M–P) Western blot analysis of AT1R expression in proteins extracted from cardiomyocytes and kidney of WT and AT1R-CKO mice under room temperature denaturation (I,K) or high-temperature denaturation (J,L) conditions, using various commercially available AT1R antibodies. Similar experiments were conducted with CHO and AT1R-overexpressing CHO stably transfected cell lines and HEK293 and AT1R-overexpressing HEK293 cells, with SDS-PAGE and exposure to five different AT1R antibodies to observe AT1R expression. RT: Room temperature; HT: High temperature.

**Figure 2:**
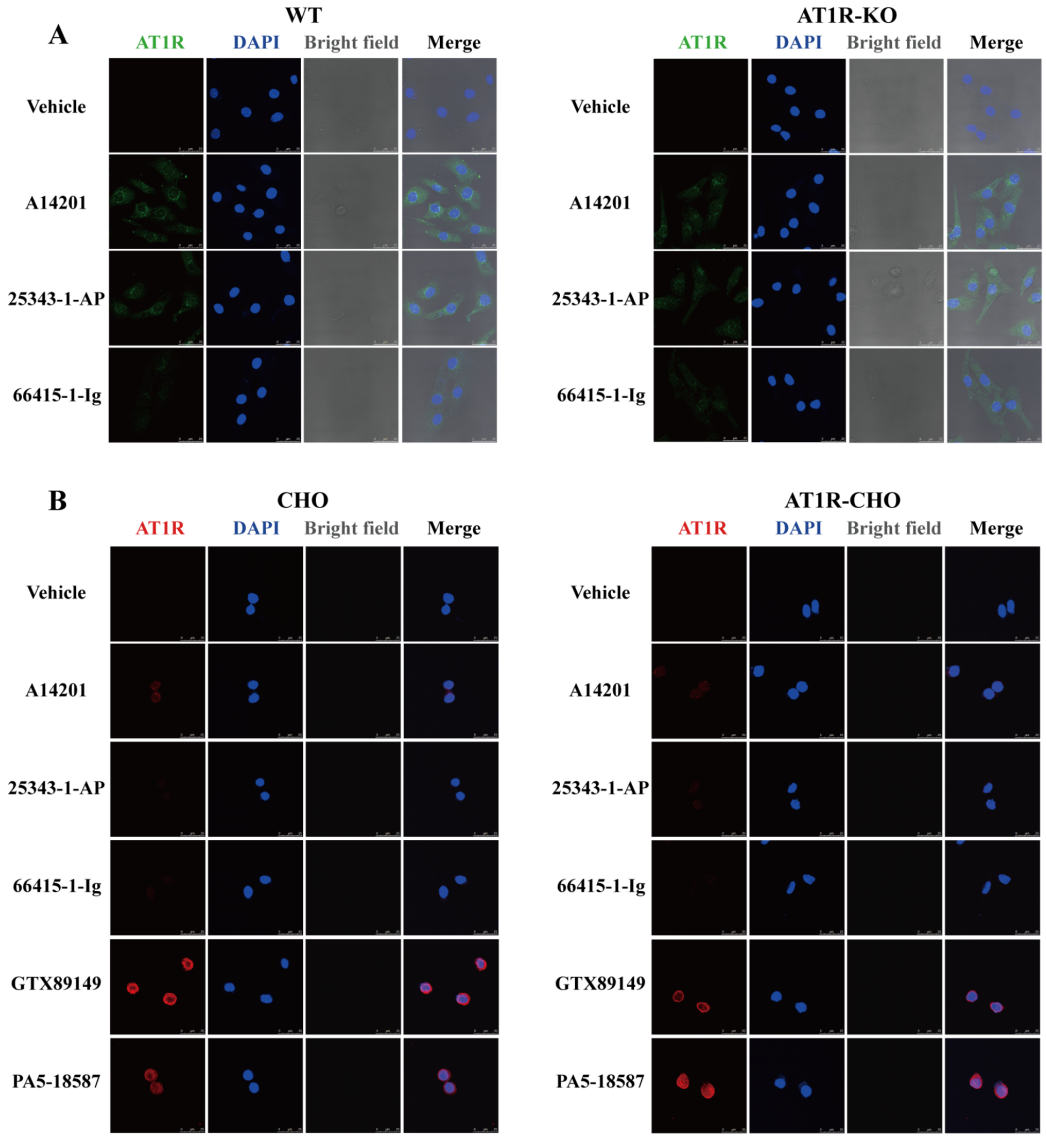
Figure 2 Lack of specificity of AT1R antibodies in immunocytochemistry assays (A,B) Immunocytochemistry staining of AT1R-KO cardiomyocytes and AT1R-CHO cells using AT1R antibodies (A14201, 25343-1-AP, 66415-1-Ig, GTX89149, and PA5-18587). Scale bar: 25 μm.

## Supplementary Data

Supplementary data is available online at *Acta Biochimica et Biophysica Sinica*.

## Supporting information

Supplementary_Materials_and_Methods

Supplementary_tables

## References

[1] Roscioni S S, Heerspink H J, de Zeeuw D. The effect of RAAS blockade on the progression of diabetic nephropathy.
Nat Rev Nephrol 2014, 10: 77–87. https://doi.org/10.1038/nrneph.2013.251.

[2] de Gasparo M, Catt K J, Inagami T, Wright J W, Unger T. International union of pharmacology. XXIII. The angiotensin II receptors.
Pharmacol Rev 2000, 52: 415–472. https://pubmed.ncbi.nlm.nih.gov/10977869/.

[3] Clark CR, Khalil RA (2024). Regulation of vascular angiotensin II type 1 and type 2 receptor and angiotensin-(1–7)/MasR signaling in normal and hypertensive pregnancy. Biochem Pharmacol.

[4] Bhullar SK, Dhalla NS. Angiotensin II-induced signal transduction mechanisms for cardiac hypertrophy.
Cells 2022, 11: 3336. https://doi.org/10.3390/cells11213336.

[5] Zucker IH, Xiao L, Haack KK. The central renin-angiotensin system and sympathetic nerve activity in chronic heart failure.
Clin Sci 2014, 126: 695–706. https://doi.org/10.1042/CS20130294.

[6] Hafko R, Villapol S, Nostramo R, Symes A, Sabban EL, Inagami T, Saavedra JM (2013). Commercially available angiotensin II At2 receptor antibodies are nonspecific. PLoS One.

[7] Herrera M, Sparks MA, Alfonso-Pecchio AR, Harrison-Bernard LM, Coffman TM. Lack of specificity of commercial antibodies leads to misidentification of angiotensin type 1 receptor protein.
Hypertension 2013, 61: 253–258. https://doi.org/10.1161/HYPERTENSIONAHA.112.203679.

[8] Bhullar SK, Dhalla NS (2024). Adaptive and maladaptive roles of different angiotensin receptors in the development of cardiac hypertrophy and heart failure. Can J Physiol Pharmacol.

[9] Sasamura H, Hein L, Krieger JE, Pratt RE, Kobilka BK, Dzau VJ (1992). Cloning, characterization, and expression of two angiotensin receptor (AT-1) isoforms from the mouse genome. Biochem Biophys Res Commun.

[10] Tabll AA, Shahein YE, Omran MM, Hussein NA, El-Shershaby A, Petrovic A, Glasnovic M,
*et al*. Monoclonal IgY antibodies: advancements and limitations for immunodiagnosis and immunotherapy applications.
Ther Adv Vaccines Immunother 2024, 12: 25151355241264520. https://doi.org/10.1177/25151355241264520.

