# Supplementary material for: Validation of six commercially available angiotensin II type 1 receptor antibodies: AT1R antibody validation
Source: Acta Biochim Biophys Sin (Shanghai). 2025 Jan 7;57(5):851–5. doi: 10.3724/abbs.2024199 (PMC12130704; doi:10.3724/abbs.2024199)
Supplement: Supplementary_Materials_and_Methods [file Supplementary_Materials_and_Methods.docx]

**Supplementary Materials and Methods**

**Animals**

*AT1R* global knockout (*AT1R*-KO) and cardiomyocyte-specific *AT1R* knockout (*AT1R*-CKO) mice were generated via the CRISPR/Cas9 system and Cre-loxP technique, respectively. All protocols were approved by the Animal Ethics Committee of Capital Medical University, Beijing, China (Ethics Nos. AEEI-2018-049 and AEEI-2020-004). Two-month-old *AT1R* global knockout Sprague‒Dawley (SD) rats and cardiomyocyte-conditioned *AT1R* knockout C57BL/6N mice were obtained from Cyagen US, Inc. (Guangzhou, China). Healthy two-month-old SD rats and C57BL/6N mice were purchased from Beijing Vital River Laboratory Animal Technology Co., Ltd (Beijing, China). All the animals were housed in a specific pathogen-free environment at the Animal Center of Capital Medical University, maintained at 18–22°C and 40%–70% humidity, with a 12/12-h light/dark cycle, and provided with free access to food and water. Neonatal rats (0–3 days old) were used for cardiomyocyte extraction experiments without rearing.

**Ligand-receptor binding assay**

Total protein was extracted from thoracic aortic tissue. Vascular tissues (50 mg) from wild-type and *AT1R*-knockout rats were homogenized with 200 μL of protein lysis buffer and ground with a plastic rod for 1 min. Magnetic beads were added for tissue fragmentation, followed by ultrasonic lysis and centrifugation at 12,000 *g* for 15 min at 4°C. ^125^I-Ang II was added to vascular tissue proteins from both wild-type and *AT1R*-knockout rats, and the binding of Ang II to AT1R was quantified.

**Extraction of primary neonatal rat cardiomyocytes**

Neonatal SD rats (0–3 days old) were sterilized in 75% alcohol. The thoracic cavity was opened via ophthalmic scissors and forceps to extract the heart, which was then placed in penicillin vials with 3 mL of pre-cooled PBS. The hearts were minced with ophthalmic scissors, digested with a solution of trypsin and collagenase, and shaken at 37°C for 5 min. The digestion process was repeated several times, and the medium was filtered through a 100-μm filter and centrifuged at 1000 *g* for 5 min. The cells were incubated in a CO_2_ incubator for 3 h for differential adhesion and then plated into 48-well plates (225 μL/well). Cardiomyocytes were observed beating in the incubator after 24 h and were further incubated for another 24 h after the medium was refreshed. The cells were stimulated with various concentrations of Ang II for 0, 5, or 10 min.

**Immunocytochemistry assay**

Primary neonatal cardiomyocytes from *AT1R*-knockout SD rats and AT1R-overexpressing CHO cell lines were cultured on coverslips and incubated at 37°C for 24 h. The cells were fixed with 4% paraformaldehyde at room temperature for 15 min, permeabilized with 0.1% Triton X-100, and blocked with 1% BSA at 37°C for 1 h, and then incubated overnight at 4°C with AT1R antibody (dilution: 1:250, the common dilution ratio was selected for different antibodies). After being washed with PBS, the cells were incubated with a fluorescent secondary antibody (1:1000) at 37°C for 1 h. Coverslips were mounted with DAPI-containing mounting medium and observed under a fluorescence microscope.

**Western blot analysis**

Tissues (50 mg) from the heart, blood vessels, liver, and kidneys of wild-type and *AT1R*-conditional knockout mice and global knockout rats were added to lysis buffer (RIPA), which was split on ice with an ultrasonic homogenizer, followed by centrifugation at 12,000 *g* for 15 min at 4°C. The protein concentration was determined using the BCA kit (Thermo Fisher Scientific, Waltham, USA). Protein extracts were subjected to either room temperature (25°C, 10 min) or high-temperature (99°C, 10 min) denaturation, separated by 10% SDS-PAGE, and transferred to PVDF membranes. The membranes were blocked with 5% skim milk and incubated with primary antibodies (dilution: 1/1000; to eliminate the differences between antibodies, we used the cross concentration in accordance with the antibody manual for elimination) overnight at 4°C (**Supplementary Table S1**). After washing, the membranes were incubated with the corresponding HRP-conjugated secondary antibodies (1:2500) for 1 h at room temperature and detected using chemiluminescence reagent.

**RT-PCR analysis**

Total RNA was isolated from the rat heart, vasculature, liver, kidney, and mouse cardiomyocytes using Trizol reagent (Invitrogen, Carlsbad, USA). RNA was extracted with the Tissue RNA Extraction Kit (ESScience, Beijing, China) and reverse transcribed into cDNA using a Reverse Transcription Kit (Thermo Fisher Scientific). The PCR mixture contained 12.5 μL of SYBR Green PCR Master Mix, 8.5 μL of ddH_2_O, 2 μL of cDNA, and 1 μL each of the upstream and downstream primers (**Supplementary Table S2**). Amplification was performed at 95°C for 15 min, followed by 45 cycles of 95°C for 20 s and 60°C for 60 s. AT1R mRNA expression was quantified.

**Statistical analysis**

Data are expressed as the mean±SD. Comparisons between two groups were performed via the Student’s *t* test, whereas data involving two variables were analyzed using two-way ANOVA. All the statistical analyses were conducted using GraphPad Prism 8.0.2. *P*<0.05 was considered statistically significant.
