# Supplementary material for: Validation of six commercially available angiotensin II type 1 receptor antibodies: AT1R antibody validation
Source: Acta Biochim Biophys Sin (Shanghai). 2025 Jan 7;57(5):851–5. doi: 10.3724/abbs.2024199 (PMC12130704; doi:10.3724/abbs.2024199)
Supplement: Supplementary_tables [file Supplementary_tables.docx]

**Supplementary Table S1. Information for six commercially available AT1R antibodies**

| **Supplier** | **Host** | **Cat No.** | **Class** | **Dilution** | |
| --- | --- | --- | --- | --- | --- |
| ABclonal | Rabbit | A14201 | Monoclonal | WB-1/1000 | IHC-1/250 |
| Proteintech | Rabbit | 25343-1-AP | Polyclonal | WB-1/1000 | IHC-1/250 |
| Proteintech | Mouse | 66415-1-Ig | Monoclonal | WB-1/1000 | IHC-1/250 |
| Thermo | Goat | PA5-18587 | Polyclonal | WB-1/1000 | IHC-1/250 |
| GeneTax | Goat | GTX89149 | Polyclonal | WB-1/1000 | IHC-1/250 |
| Abcam | Rabbit | ab124505 | Polyclonal | WB-1/1000 | IHC-1/250 |

**Supplementary Table S2. Sequences of primers used in this sutdy**

| Gene | Species | Forward primer (5′→3′) | Reverse primer (5′→3′) |
| --- | --- | --- | --- |
| *AT1R* | Mouse | ACTCACAGCAACCCTCCAAG | ATCACCACCAAGCTTTTTCC |
|  | Rat | TAACCAAGCAAAGCCGTCTTG | TCACCTCTGACTGTGTACATTTCTCA |
| *β-actin* | Mouse | GATGGTGGGAATGGGTCAGAAGG | TTGTAGAAGGTGTGGTGCCAGATC |
|  | Rat | CTATCGGCAATGAGCGGTTCC | GCACTGTGTTGGCATAGAGGTC |
